# Hospital Unification in Cape Town: The Method of Control

**Published:** 1915-06-19

**Authors:** 


### HOSPITAL UNIFICATION IN CAPE TOWN.

### The Method of Control.

THE Cape Hospital Board, which came into existence about three years ago as the result of the passing of the Cape Hospitals and Charitable Institutions Ordinance, is now in a better position to report upon the results of the movement in hospital unification set in motion by the Legislature.

From the second annual report we learn that the institutions controlled by the Board are now eight (five hospitals, one dispensary, and two convalescent homes). The hospitals have an aggregate number of available beds of 366, but at the Somerset Hospital—the largest unit—107 additional beds have been provided by making temporary use of a new nurses' home, a lecture hall, and other parts of the building, the Board having, for war purposes, undertaken to find proper hospital accommodation for military patients up to a maximum of 223.

At the hospitals last year 4,200 in-patients were accommodated; at the convalescent homes 500 cases were received, and over 30,000 attendances of out-patients were recorded at the institutions within the group. Of the in-patients approximately 1,200 were cases for whose maintenance payment at varying rates was made.

The Board consists of thirty-eight members, a proportion being elected respectively by contributors, local authorities, the Administrator, and the hon. visiting medical officers. Each unit has, in addition, its own committee of management. The method of control by the committees of the several institutions has been a vexed quesion since the Board came into being, and but for the exercise of much forbearance and tact on both sides would have led to considerable friction, with consequent impairment of efficiency. A large measure of self-government has been granted to the committees, but there are those on the Board who advocate greater centralisation, particularly in the book-keeping and secretarial work of the institutions. It has probably been found that in South Africa, as in this country, individuality in an institution is often a great power for good, and, very wisely, the Cape Board have decided to go about the settlement of this difficult question leisurely, taking into their counsel those who are best acquainted with local needs and peculiarities.

There have, however, been found methods of correlation which could be proceeded with at once, such as the admission, tuition, and practical training of nurses and the grading of payment and clothing of the domestic and nursing staffs.

Soon after the outbreak of the war the Board was notified by the Provincial Administration that it would be necessary to curtail, as far as possible, all expenditure in which State funds were involved, and that it would not be possible to allow the Board to undertake any new commitments in respect of building operations which would necessitate any further issue of loan moneys. The Board, however, were allowed to proceed with the new pavilion at the Somerset Hospital, the Nurses' Home at the Victoria Cottage Hospital, and some minor alterations to other buildings. The financing of important schemes of building is provided for by a system of loan from the Provincial Administration, the last interest arranged being at the rate of 4 per cent. per annum, with an annual charge for redemption in forty years.

Powers are being sought to commence a scheme for granting pensions or otherwise to make provision for officers and employees upon retirement through age or loss of health.

The average cost per in-patient in 1914 ranged from 4s. 10d. to 8s. 9d. per day; Somerset and Wynberg Hospitals, which received most cases, showed respectively an average of 5s. 10d. and 5s. 8d. With the exception of one small institution there was a substantial reduction in cost all round as compared with the previous year.

Mr. W. Sinclair Thompson, the Secretary and Treasurer, must be congratulated upon the production of this report in such good time. It is a well-arranged volume of ninety-three pages, showing in detail the accounts and subscription lists of each of the institutions represented by the Board. The report would be improved by the publication of a short summary of the provisions of the ordinance under which the Board was constituted.

